# Supplementary material for: Effect of Red Light on the Expression of the Phytochrome Gene Family and the Accumulation of Glycoside Alkaloids in Potatoes
Source: Foods. 2023 Nov 21;12(23):4194. doi: 10.3390/foods12234194 (PMC10706466; doi:10.3390/foods12234194)
Supplement: Supplementary file 1 [file foods-12-04194-s001.zip › foods-2666623-supplementary.pdf]

Table S1. Primer design of *StPHY.*

Primer name	Primer sequence
PHYA-Fw	5′ACATTGTGCGGCTCTACT3′
PHYA-Rv	5′TTTCTCCTTCTTCATCCC3′
PHYB-Fw	5′AGTTGTTGTCGGCTCA3′
PHYB-Rv	5′GGACTGGCTATCTGTATG3′
PHYB2-Fw	5′GGGATGGATGAACTGAG3′
PHYB2-Rv	5′ACCAACGACTTCCCTAT3′
PHYC-Fw	5′AAGGCAAGGAGGAGAAA3′
PHYC-Rv	5′GAGATGGACTGCGGATA3′
PHYE-Fw	5′ATTGATTGAGACAGCCACT3′
PHYE-Rv	5′TTCCTCCTCACCTAACAGA3′

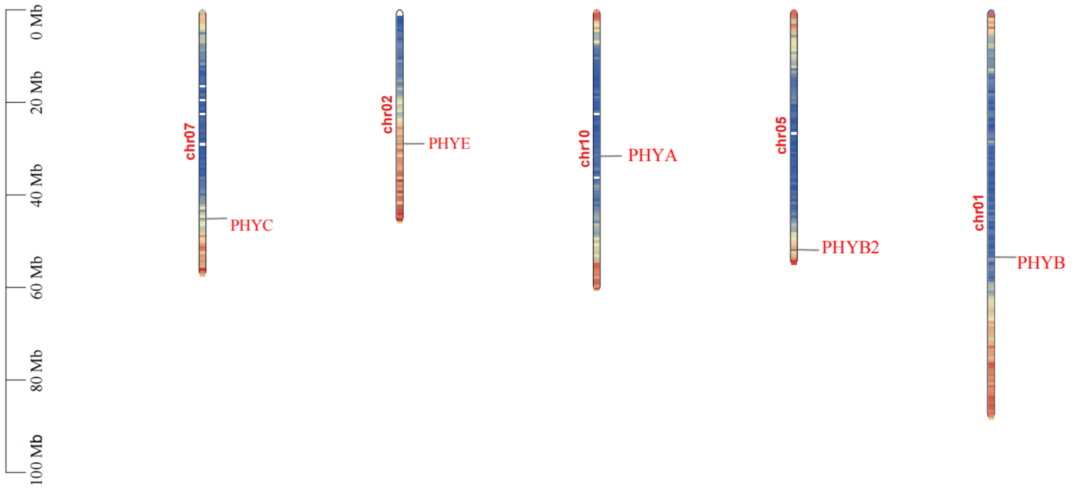

Figure S1. Chromosome localization analysis of PHY.

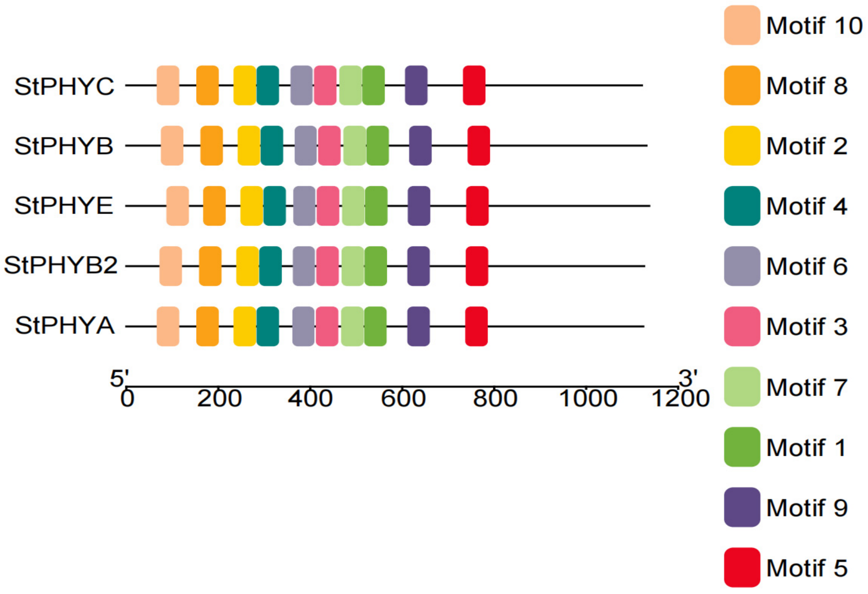

Figure S2. Motif analysis of PHY.
